# Minimization of Energy Losses in the BLDC Motor for Improved Control and Power Supply of the System under Static Load

**DOI:** 10.3390/s22031058

**Published:** 2022-01-29

**Authors:** Andrzej Sikora, Adam Zielonka, Marcin Woźniak

**Affiliations:** 1 Faculty of Electrical Engineering, Silesian University of Technology, 44-100 Gliwice, Poland; andrzej.sikora@polsl.pl; 2 Faculty of Applied Mathematics, Silesian University of Technology, 44-100 Gliwice, Poland; adam.zielonka@polsl.pl

**Keywords:** BLDC motor, power electronic, saved energy

## Abstract

In this article we present the optimal method of controlling and supplying a BLDC motor under static load, proposed and implemented as a result of the research. A research infrastructure was developed to measure and analyze variants of the motor control. In the research we determine possible losses of electric energy released in the form of heat in the tested engine elements. The test results showed that the lowest energy losses are provided by the variant where the control signals are obtained from an external magnetic disc and the motor is powered by an additional DC/DC converter. The conclusions from the analyses allowed for the selection of the best variant of motor control and power supply, which minimizes energy losses during the BLDC motor operation.

## 1. Introduction

Brushless Direct-Current Motors (BLDC) are characterized by very high efficiency, which in the case of the construction of energy-saving drives, in particular powered by autonomous energy sources, is very important. There are many important conclusions for future trends [1]. In particular, the construction of the electronic commutator is interesting [2]. Two variants of sensors determining the position of the motor shaft have been proposed and compared in [3]. The research determines which of the examined variants are more favorable in terms of energy losses for optimal operation.

Classic electric motors can be divided into two groups, due to the way they are powered:

- AC powered motors. These motors are powered from a three-phase power grid, where small loads are powered by single-phase, while high power loads are constructed as three-phase receivers. Three-phase alternating current motors can be categorized as synchronous motors and asynchronous motors;
- DC-powered motors. These motors were previously obtained from rotating converters such as the Leonard system. Nowadays these motors are powered using static transformers, that is, diode or thyristor rectifier systems.

Both in the first and the second group of motors that have been used for a long time, both the stator and the rotor had winding (excluding small motors, e.g., in toys), where one winding acted as the excitation winding. Such models were to generate a magnetic flux, and the other was a winding armature [4]. The invention of strong rare earth magnets gave engine designers new possibilities. It is possible to eliminate winding generating the magnetic field and in its place the installation of permanent magnets [5,6]. Thanks to this design, the current flow is not needed to generate the magnetic field (no excitation winding), it eliminates energy losses in the excitation winding automatically [7,8]. The use of strong magnets to excite electrical machines allows for the construction of more efficient machines [9]. Electric machines excited by permanent magnets can be divided into two groups due to the placement of magnets:

- Machines in which permanent magnets are placed on the stator. In these machines, the armature winding located on the rotor is powered by a classic mechanical commutator;
- Machines in which permanent magnets are placed on the rotor. In these machines, the non-rotating armature winding requires a power supply through the so-called electronic commutator. This part supplies the windings synchronously with the rotation of the rotor.

The second solution eliminated the mechanical commutator, which, due to its durability, limited the life of the electromachines [10,11,12]. The mechanical commutator could cause sparking, which introduced additional electromagnetic disturbances. The use of an electronic commutator increased service because correctly selected switching elements of an electronic commutator, such as power transistors, are much more durable than mechanical elements [13]. Motors with magnets placed on the rotor can be further divided into two groups:

- Brushless Direct-Current Motor (BLDC) in which the induced voltages during rotation of the rotor have a trapezoidal waveform [14];
- Permanent Magnet Sync Motor (PMSM) in which the induced voltages during the rotation of the rotor have a sinusoidal wave form [15,16] wave.

Powering motors from both groups requires the tracking of the angular position of the rotor in order to properly control the valves of the electronic commutator [17,18]. Motors with a sinusoidal distribution of the PMSM magnetic field require a sinusoidal voltage supply, which places greater demands on the electronic commutator system [19,20]. Motors with a (rectangular) trapezoidal magnetic field distribution are powered in such a way that the task of the electronic commutator is only to switch the voltage to the individual windings synchronously with the rotation of the rotor [21,22].

The main purpose of this work is to propose a new method of supplying a BLDC motor and a new sensor for determining the position of the BLDC motor shaft. As a result of our research we demonstrate that the newly proposed solutions reduce energy losses in motor components. The scope of this research work is to present a new design and construction of the entire BLDC motor power supply system. Our developed research stand is tested to draw conclusions on our developed model of construction. The research infrastructure was made based on the use of the Internet of Things, thanks to which it was possible to seamlessly collect data and process them in order to obtain computed results for interpretation. In Table 1, we have presented main advances from the application of the proposed research innovation to the construction and optimization of the BLDC motor. We can see that the presented spectrum of applications is very wide. BLDC motors are applied in power tools, ventilation, motor engines both in road vehicles and flying vessels of different size and purpose, and so forth. Therefore, the presented novel idea of construction and improvements to the positioning of BLDC motor features are very important aspects of this research.

## 2. Developed Research Model

**Figure 1:**
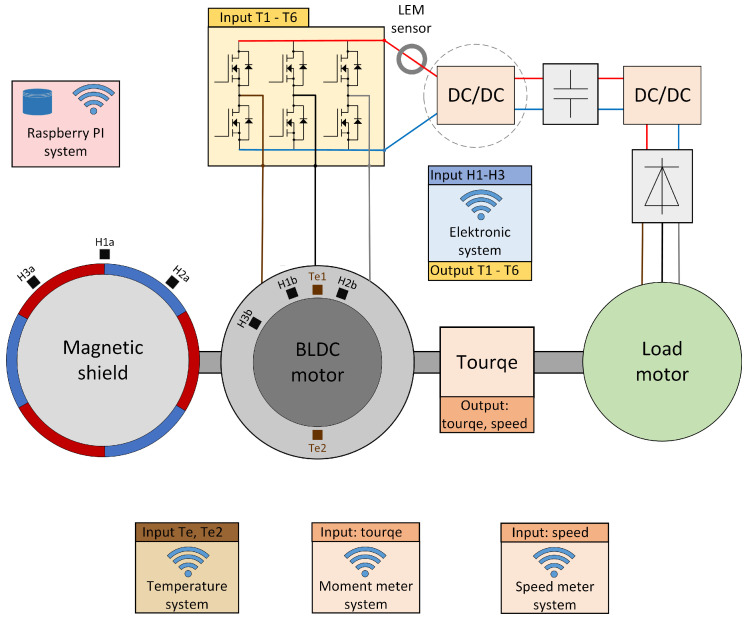
Scheme of the construction of the BLDC engine research stand with the load and marked measuring apparatus with sensors used to measure all features like speed, momentum (torque), temperature.

As part of the work, the BLDC research engine was composed and examined. Various variants of the motor power supply and various sensors for determining the angular position of the motor shaft were tested. The tests used a three-phase electronic commutator bridge system with an optional DC/DC converter and an electronic commutator system. Both of these systems were designed and created as part of our research work. The scheme of the test stand with the installed measuring infrastructure is shown in Figure 1.

**Figure 2:**
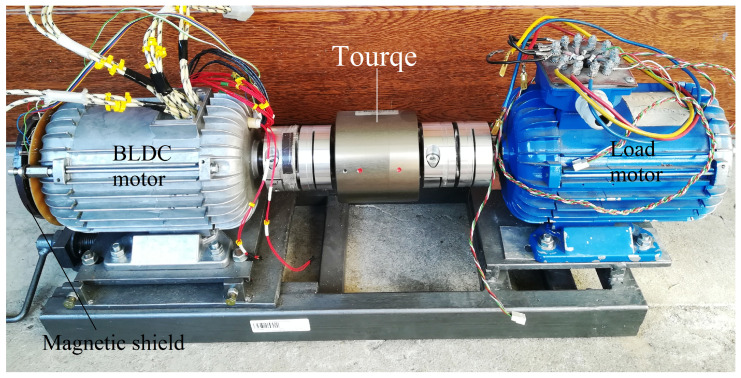
An image showing our developed research stand model.

The basic element of the test stand is a BLDC motor connected by a shaft to a three-phase loading machine excited by permanent magnets. The shafts of the motors are connected with a torque meter via clutches, which are shown in Figure 2. The tested motor is equipped with additional measuring windings located in the stator and a set of hall sensors installed in the stator. These sensors are used optionally to determine the angular position of the rotor. The shafts of both machines were connected via Dataflex 22/20 torque gauge in order to measure the load of the tested engine. An additional external sensor (developed by us as a part of research) is attached to the motor shaft, which allows us to determine the angular position of the rotor, described as a Magnetic shield. Optionally, this sensor can be replaced with a 10-bit MAL 405-PA encoder, the output signal of which is in the form of a Gray code. The motor is powered by an electronic commutator with T1-T6 transistors as switching elements. The control is based on the ATtiny microcontroller. Optionally, signals from sensors determining the angular position of the rotor are supplied to the microcontroller. On the other hand, the output signal is the information which of the bridge transistors is to be driven. The stand is built in a reverse operation system, that is, electric energy obtained from the loading machine after rectification by a rectifier system consisting of six diodes and after processing by a DC/DC converter, is transferred to the battery bank from which the BLDC motor is powered. Thanks to this solution, only the energy needed to cover losses in the test stand is taken from the power grid.

**Figure 3:**
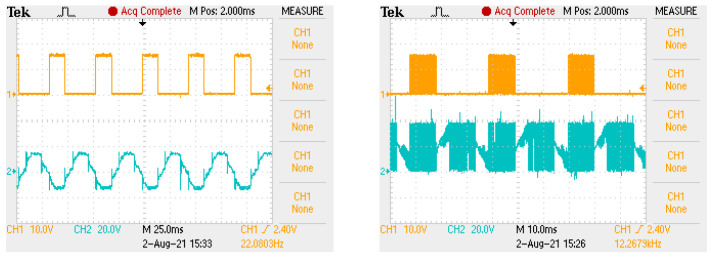
Oscillograms showing the control signal of the bridge transistor (**left**) and the output voltage of the transistor for BLDC motor control with and without PWM (**right**).

The developed research stand allows us to optionally power the engine in two ways. The first is that the transistors of the electronic commutator bridge are actuated only on the basis of unmodified signals provided by the rotor angular position sensors. The second of the tested variants of motor power supply is that the transistors of the electronic commutator bridge are actuated on the basis of the signal from the shaft position sensors modified by the PWM signal. Examples of the control voltage waveforms (orange) fed to the transistor gate and the voltage on the motor windings (blue) in the case of both power supply variants are shown in the oscillograms in Figure 3. The left waveform shows the waveforms for the variant with DC/DC converter, and the right waveforms shows when the drive signal of the bridge transistors is modified by the PWM signal.

It is possible to change the average value of the voltage supplied to the motor windings, which allows the motor rotational speed to be regulated by changing the PWM signal duty cycle. Additionally, the duty cycle of the PWM signal enables the implementation of the current limiting function. In the first variant of the power supply, the adjustment of the engine speed and the current limitation function are performed on the basis of an additional DC/DC converter marked in the diagram Figure 1 with a broken circle. In both cases, the current limitation is based on the information signal about the value of the current consumed by the electronic commutator bridge, measured with the LEM sensor.

**Figure 4:**
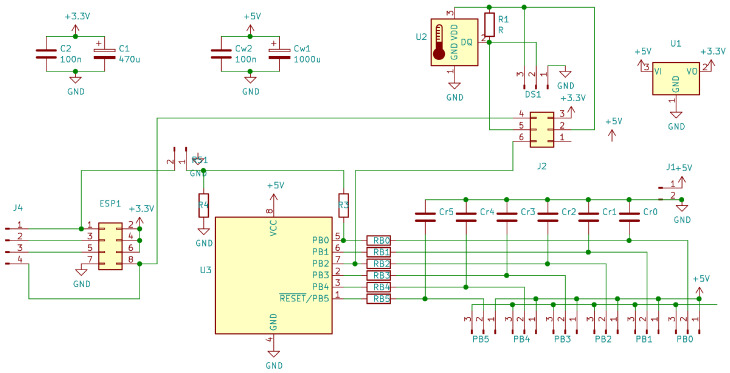
Schematic diagram of the universal measuring system applied in our research stand.

The research infrastructure of the test stand was designed based on Wi-Fi communication. The Raspberry Pi class microcomputer serves as the master server for collecting measurement data. The measurement modules are connected with the sensors built in the stand by electric wires. Each of the modules is responsible for measuring data and sending them through the ESP 8266 system to the master unit, on which the application that interprets and collects measurement data is running. All measurement modules were created on the basis of the same system (made as part of the work), essentially consisting of the ATtiny 13 microcontroller—connected to the sensor and converting the measurement data into a physical quantity value. The universal measuring system shown in Figure 4 uses the ATtiny13 microcortoler due to the fact that it is equipped with an analog-to-digital converter and has an internal counter with the possibility of selecting a prescaler.

**Figure 5:**
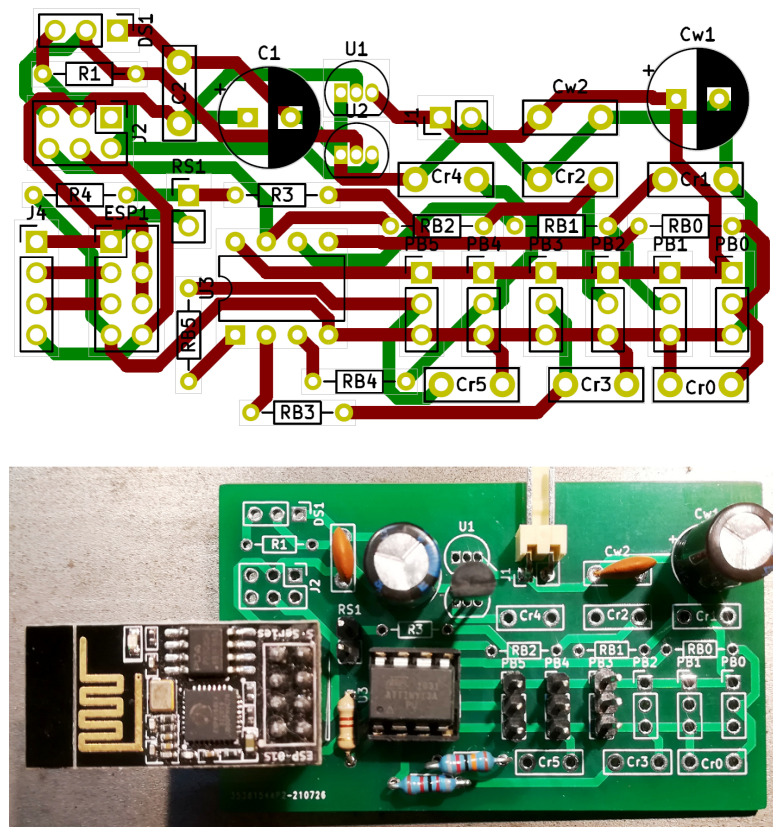
PCB design for a universal measurement system which we have developed (**top** figure) for our research and its practical implementation according to our design template (**bottom** picture).

Our developed measuring system, presented in Figure 5, uses sensors which give an analog signal at the output (a signal from thermistors representing temperature and a signal representing the load torque) or a digital signal (coming from hallotrons and rotational speed signal from a torque meter). The analog signal is fed to the internal analog-to-digital converter, while the digital signal is fed to the microcontroller’s pin signaling the interrupt. The microcontroller, using an internal counter, measures the time between successive interrupts (changes in the logical levels of the digital signal). In this way, the value of the measured physical quantity is determined for digital data. Then, via the serial link, the ATtiny microcontroller 13 transmits the determined value to the ESP 8266 chip, which transmits the data via Wi-Fi to the Raspbbery Pi operating in the AccessPoint mode.

## 3. BLDC Motor

The basic feature of brushless of DC motors (BLDC) is that the rotor excites the machine while the armature windings are located in the stator of the motor. It is the opposite design to the classic DC motor. The consequence of this design is the lack of a mechanical commutator.

**Figure 6:**
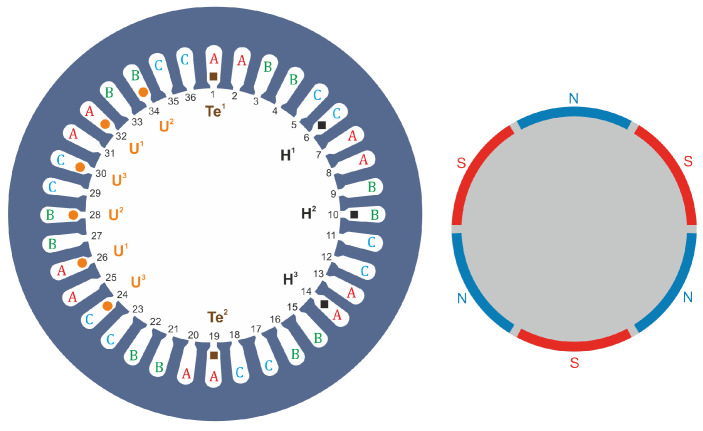
Illustrative drawing showing our developed construction of a BLDC motor: **left** figure—stator with marked windings and arrangement of Hall sensors $H^{1}$, $H^{2}$, $H^{3}$, additional windings $U^{1}$, $U^{2}$, $U^{3}$ and $Te^{1}$, $Te^{2}$ temperature sensors; **right** picture—rotor, with three pairs of magnets glued on the side surface of the rotor.

**Figure 7:**
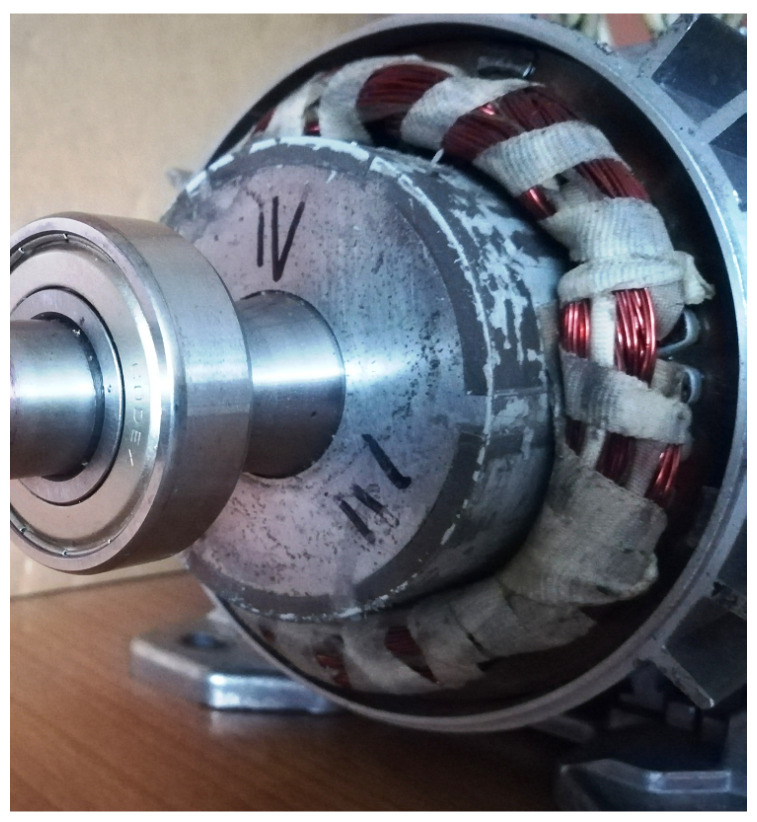
Protruding rotor of the motor, on the circumference of which we can see glued magnets separated by a slot.

The schematic diagram of the tested BLDC motor with the location of the sensors built in it is shown in Figure 6. The design of BLDC motors uses permanent rare earth magnets, which are placed on the surface of the motor rotor, as shown in Figure 6 (right picture). The rotor of the tested motor is characterized by a layer of steel between the magnets forming the rotor shaft, which can be seen in the diagram as a gray gap between the magnets. The stator of the tested motor (Figure 6, left illustration) has windings arranged symmetrically in 36 slots along the circumference, similar to three-phase asynchronous motors. As already mentioned, the excitation of the BLDC motor is provided by the rotor, unlike in the DC motor, where the excitation is provided by the winding placed on the stator. The fixed armature winding is located on the stator and requires power supply via an electronic commutator. Figure 7 shows the protruding rotor of the tested motor, with a metal gap between the magnets glued on the circumference of the rotor.

Additionally, the engine is equipped with three hall sensors $H^{1}$, $H^{2}$, $H^{3}$ of selected stator slots arranged in relation to each other so as to ensure an unambiguous control sequence. In addition to Hall effect sensors, the stator is equipped with additional windings, which provide control signals alternative to Hall sensors. The stator is also equipped with two temperature sensors—thermistors in order to analyze the motor heating (resulting from energy losses in the motor).

**Figure 8:**
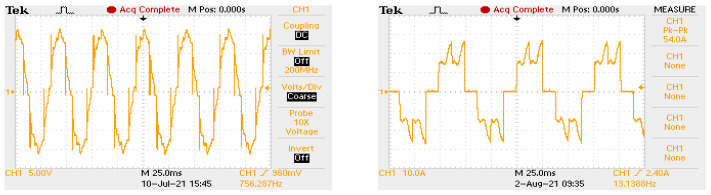
Sample research recording of the phase-to-phase voltage waveform of the tested motor (**left** oscillogram) and the current of one motor phase (**right** oscillogram).

The voltage induced in the stator windings during the rotation of the rotor theoretically follows a trapezoidal course. An example of recording the waveform of the phase voltage and the current of one phase is shown in Figure 8. For this reason, the BLDC motor power supply requires voltage switching (sequential switching) to the appropriate windings depending on the angular position of the rotor. Obtaining the torque directed in the appropriate direction (right or left) entails the necessity to set an appropriate supply sequence synchronized with the angular position of the rotor. In order to synchronize the supply sequence with the angular position of the rotor, it is necessary to use sensors that determine the angular position of the rotor. The task of the rotor angular position sensors is to determine when the next power sequence is to be switched. For a three-phase motor with one pole pair, if two windings are always powered, there are six power sequences per revolution, for motors with more pole pairs, the sequences are repeated. Signals from various sensors can be used to determine the angular position of the rotor of the motor. In our considerations, we examined three different control variants, which are characterized below.

In the first case, the signals determining the angular position of the rotor are the voltages from additional windings located in the stator slots (marked in Figure 6 as $U^{1},U^{2},U^{3}$). Due to the fact that the voltages are induced during the rotation of the motor shaft, the main disadvantage of this solution is the lack of a signal when the rotor is stopped. As a result, when starting the engine, it is impossible to determine what power sequence should be set to obtain the correct direction of rotation of the motor. With additional windings, starting the motor and then ensuring rotation in the appropriate direction can be performed by specifying a power supply sequence for a given direction of rotation.

**Figure 9:**
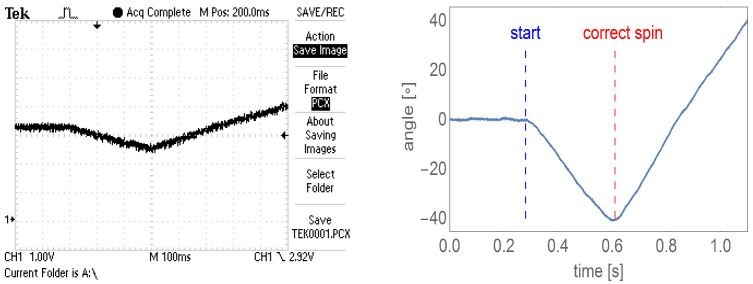
The oscillogram of the analog encoder signal recorded during the start-up of the controlled motor based on the voltages from the additional motor windings (**left** figure), the graph of the rotation angle versus time corresponding to the oscillogram, with the moment of rotor movement marked (start) and determination of the correct spin direction (correct spin) (**right** figure).

In the first stage (not having signals from additional windings yet), the electronic commutator supplies the motor according to the set control sequence for the given direction of rotation. Due to the lack of information about the initial position of the rotor, such a strategy causes the motor to make a partial rotation of the rotor in the opposite direction to the set one. Figure 9 shows the oscillogram and the graph showing the interpretation of the analog encoder signal recorded during the start-up of the motor controlled on the basis of additional windings.

In a pessimistic case, after a partial rotation in the opposite direction, the rotor of the engine will start to spin in accordance with the given sequence of powering the engine (stepper motors are supplied in a similar way). After obtaining the appropriate rotational speed, it can be controlled using voltages from additional windings. Such a control system can be used for drives insensitive to the momentary rotation of the shaft in the opposite direction than the set one, e.g., boat drive, or fan.

The second solution is control based on signals from Hall sensors located in the grooves of the engine, marked as $H^{1},H^{2},H^{3}$ in Figure 6. These hall sensors directly measure the magnetic field generated by the rotor magnets and thus always allow the determination of the appropriate control sequence.

**Figure 10:**
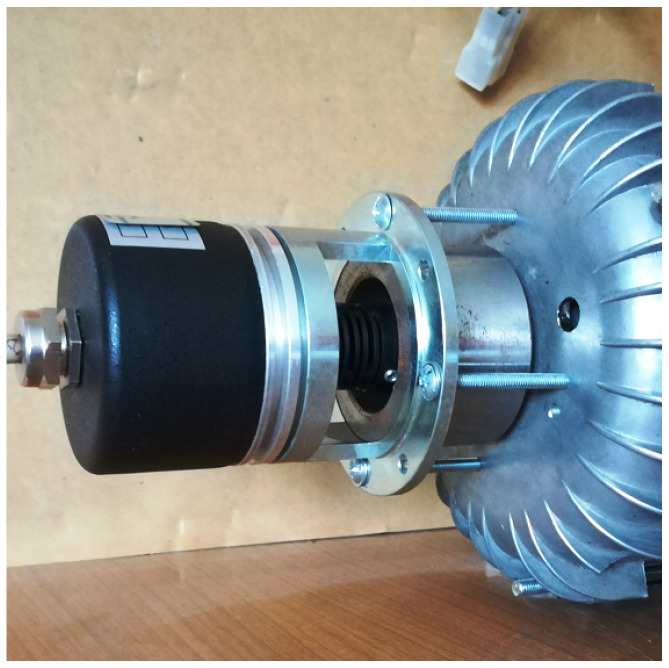
Digital encoder—optional variant of determining the position of the motor shaft.

A third alternative solution is to install an absolute encoder (analog or digital) on the motor shaft (Figure 10). Sensors of this type are sensors that determine the precise angular position of the rotor. Precise determination of the angular position of the rotor is necessary when supplying PMSM motors. In the case of BLDC motor power, the information provided by the encoder is redundant since only a few motor shaft positions are required to control the BLDC motor.

**Figure 11:**
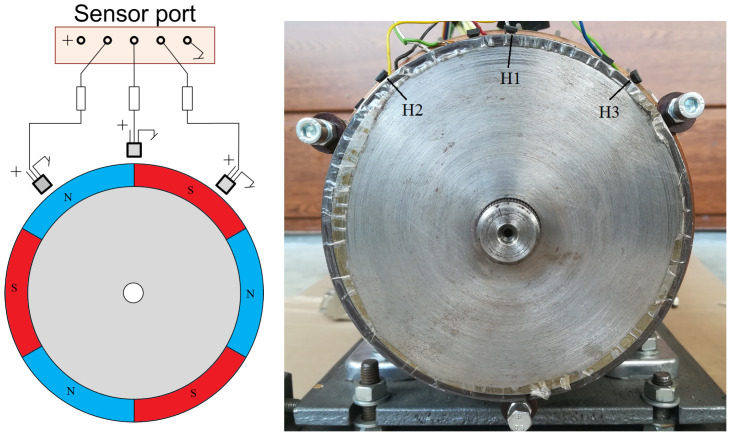
External sensor of the motor shaft position in schematic model (**left** figure) and our implementation (**right** figure).

The fourth optional control method is an external magnetic sensor mapping the rotor poles, cooperating with hall sensors. As part of the research, the authors made such a sensor, the diagram and photo of which are shown in Figure 11. This sensor has the form of a large (comparable to the size of the motor body) magnetic disk imitating the distribution of the magnets in the rotor of the motor. The large radius of the blade allows for precise switching of successive control states.

The first of the proposed methods of determining the position of the motor shaft has significant limitations due to the specificity of the engine start-up. Comparing the other three solutions, the use of an encoder ensures precise determination of the position of the motor shaft, but at the same time significantly increases the cost of the motor power supply system.

Solutions based on the hall sensor are sufficient to determine the position of the motor shaft necessary for its control. The main advantage of these solutions has a significantly lower cost than when using an encoder.

An additional advantage of using an external hall sensor is the possibility of repairing it without interfering with the engine structure, which would be necessary in the event of damage to the hall sensor installed in the engine groove. In the further part of the work, a comparative analysis of the engine operation will be carried out depending on the choice of the engine control method, the variant with Hall effect sensors installed in the engine and the variant with an external magnetic disc.

## 4. BLDC Motor Power Supply

The lack of a mechanical commutator in the construction of a BLDC motor requires the creation of an electronic commutator. The power supply of the BLDC motor requires sequential switching of power electronic keys strictly dependent on the position of the motor shaft and the direction of rotation. Failure to meet the above requirement: significant delay or advance (by two control sequences) of switching in relation to the rotating rotor or driving the transistors for the direction opposite to the rotation of the motor shaft causes the flow of very high current through the open transistors, which leads to their destruction. Providing energy-saving power requires precise switching of the power transistors of the electronic commutator and thus precise determination of the angular position of the motor shaft. More precise detection characteristic positions of the motor rotor result from the position of the rotor magnets. First, the construction of the transistor bridge and electronic commutator system will be discussed, followed by the control method.

**Figure 12:**
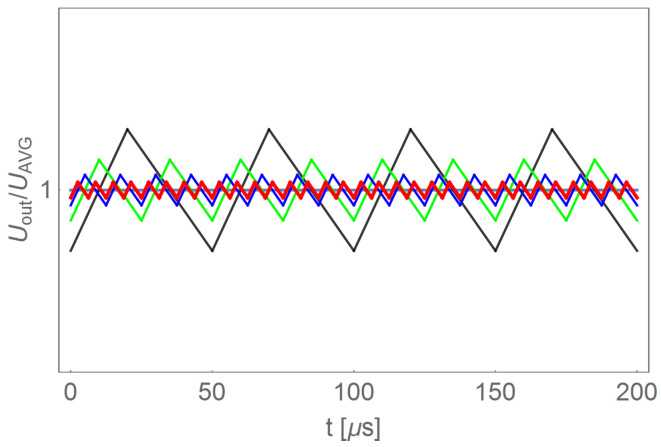
Simulated example waveforms of the supply voltage of an electronic commutator in a system with the use of an additional DC/DC converter for four different filters.

The DC/DC converter has been used to lower the voltage from the battery bank to the required level. Regulation of the output voltage is based on the PWM signal control. A filter reducing the pulsation of the output voltage is applied at the converter’s output. The pulsation of the output voltage depends on the filter time constant and the switching frequency of the PWM transistors. The pulsation reduction can be achieved by selecting a suitably large capacitor and coil or by increasing the PWM frequency. Examples of voltage waveforms are shown for four different PWM frequencies in Figure 12. In order to limit the size of the filter elements, high-frequency control was used.

The novel BLDC motor system constructed in our research allows for speed and load regulation based on the use of pulse width modulation (fast PWM) using an 8-bit counter. This approach allows us to obtain 256 different values of average voltage at the output of the system. This resolution is sufficient to perform our developed computer simulation model for BLDC motor applications presented in Table 1.

**Figure 13:**
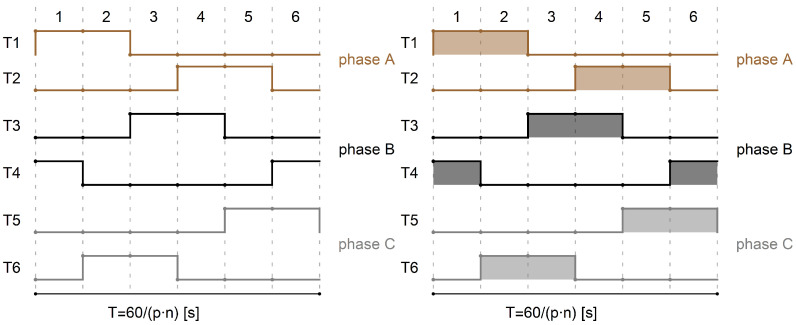
Waveforms of signals controlling the gates of the electronic commutator bridge transistors for the variant with an additional DC/DC converter (figure on the **left**), for the variant without an additional DC/DC converter (figure on the **right**).

**Figure 14:**
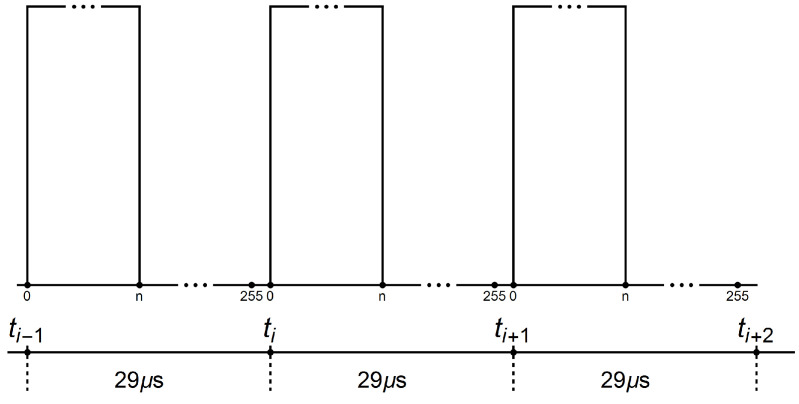
The course of the signal controlling the transistors of the electronic commutator bridge for the variant without an additional DC/DC converter.

**Figure 15:**
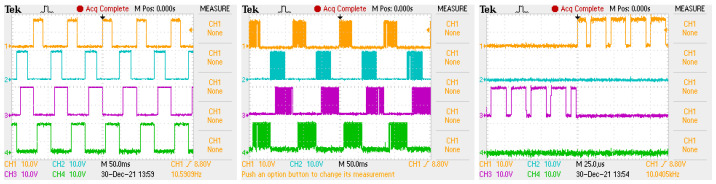
Oscillogram showing transistor control signals in case of a non-PWM power supply with a time base of 50 ms (**left** picture), in case of a PWM power supply with a time base of 50 ms (**middle** picture) and a power supply with PWM modulation with a time base of 25 μs (**right** picture), where in all figures orange is for the transistor $T_{1}$, blue for the transistor $T_{2}$, purple $T_{3}$ and green for $T_{4}$.

Figure 13 shows the signals controlling the transistors of the electronic commutator bridge for two variants of power supply and motor speed control. The waveforms in the figure on the left are for a system with a DC/DC converter, and in the figure on the right, for a system without a converter. In the second case, the shaded fields visible in Figure 13 mean that the waveform is modulated with a PWM signal. Sample results are visible in close-up form in Figure 14. In both cases of electric supply, pairs of transistors supplying one motor phase ($T_{i}$ i $T_{i+1}$, for i=1,3,5) are controlled in such a way that after switching off the transistor of a given phase, switching on the next one takes place only after one control tact. For example, in Figure 13, the $T_{1}$ transistor is turned off after the 2nd measure of the sequence consisting of 6 measures (marked with the numbers 1,…,6 in the upper part of the figure), while the $T_{2}$ transistor is turned on in the 4th control step. This solution prevents the occurrence of short circuits in the branches of the electronic commutator bridge. It can be assumed that one control cycle is the dead time between switching transistors of a given bridge branch. Figure 15 shows the recorded control waveforms of four transistors supplying two phases of the electronic commutator bridge, where the left figure concerns control with the use of a DC/DC converter, the middle one—without a DC/DC converter—and the right one shows the PWM signal supplied to the bridge transistors in a system without a DC/DC converter.

### 4.1. Construction of an Electronic Commutator

**Figure 16:**
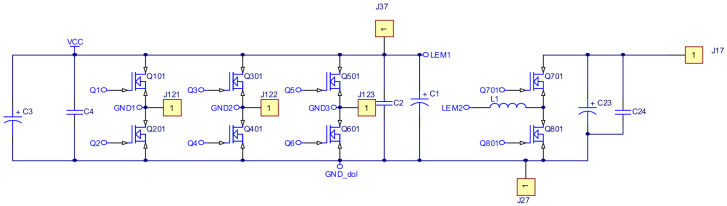
Schematic diagram of the power elements of the electronic commutator bridge and DC/DC converter.

**Figure 17:**
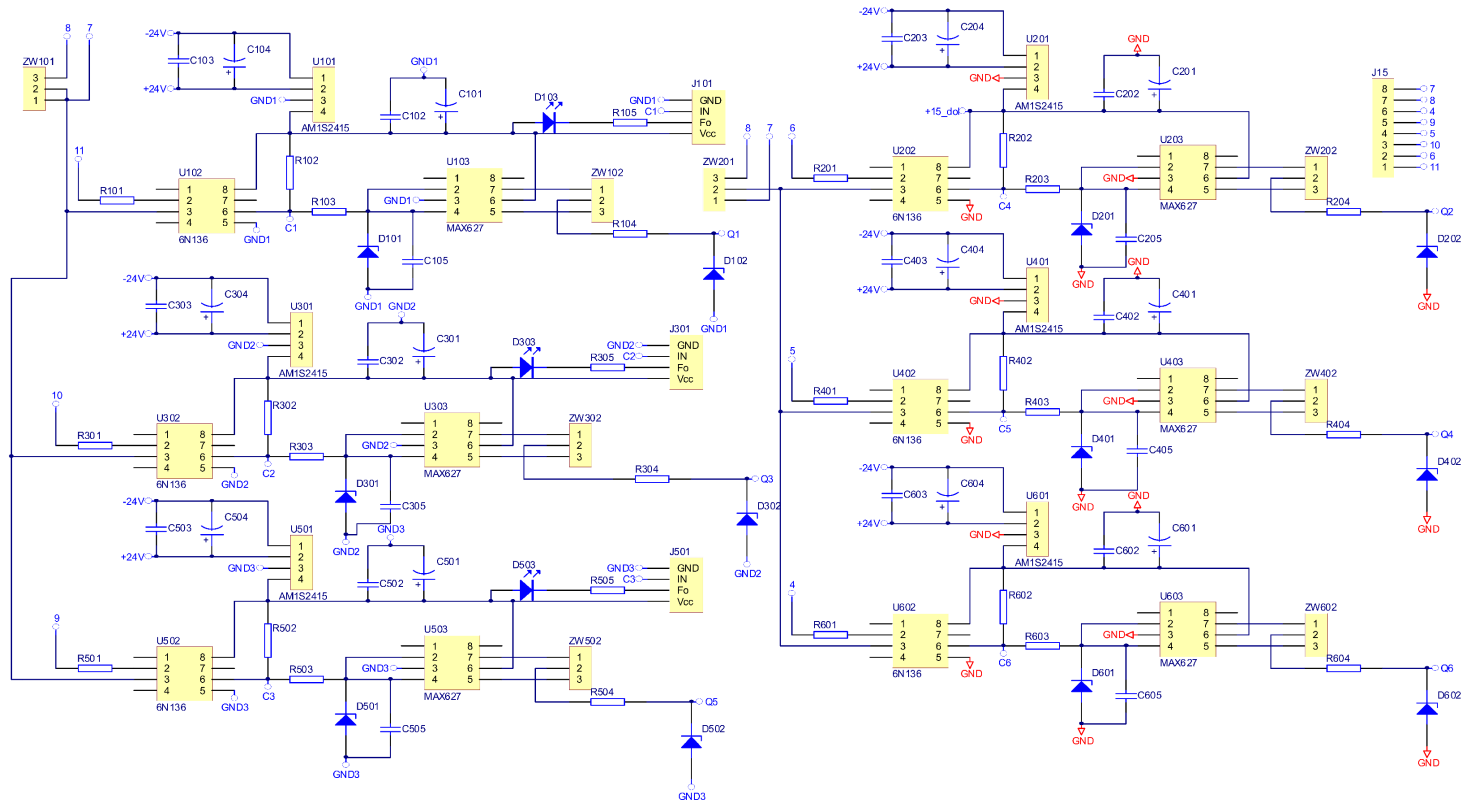
Schematic diagram of the construction of a rectifier bridge.

**Figure 18:**
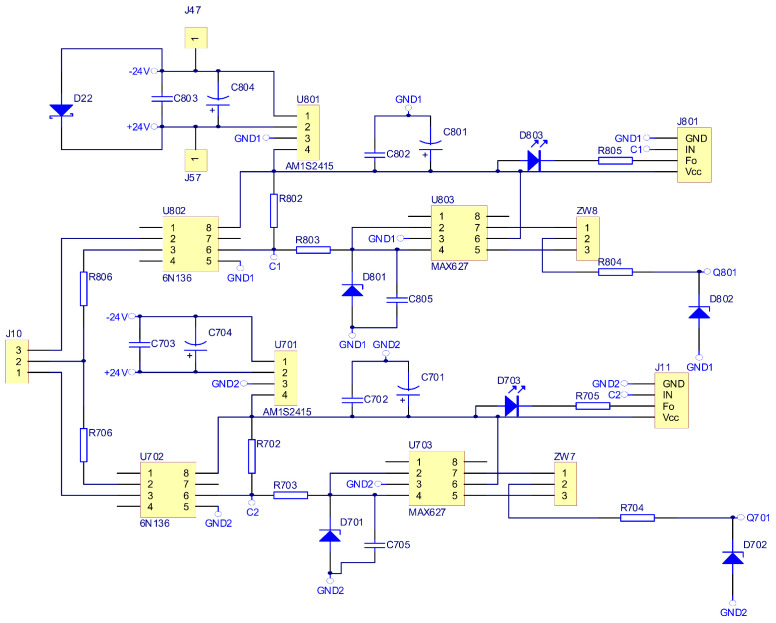
Schematic diagram of the DC/DC converter construction.

As part of the research work, an electronic commutator system was created. The schematic diagrams in Figure 16, Figure 17 and Figure 18 show the elements of the electronic commutator bridge and their connections. Figure 18 shows the control elements of the DC/DC converter.

**Figure 19:**
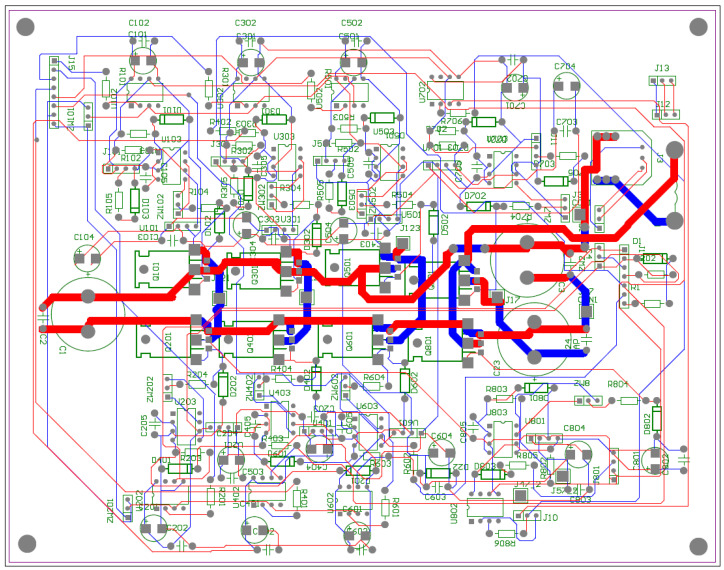
Electronic commutator circuit board design.

**Figure 20:**
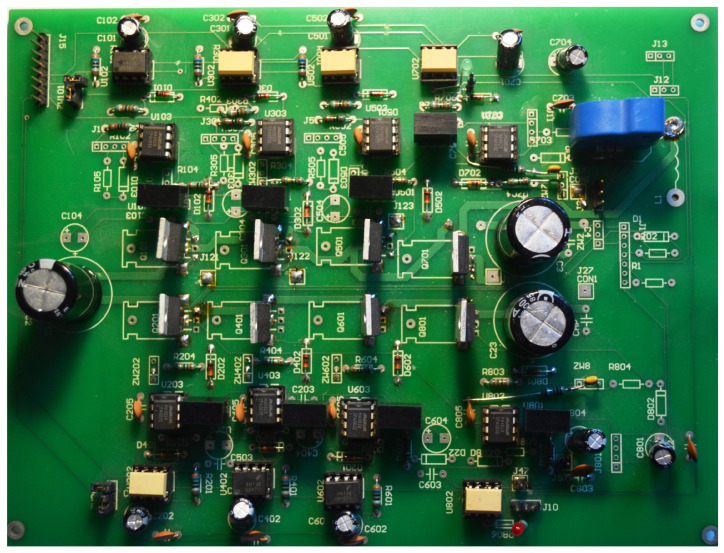
Developed BLDC motor control circuit board without the heat sinks on the transistors.

Figure 16 includes six electronic commutator bridge transistors labeled Q101–Q601, and DC/DC converter transistors Q701 and Q801. Between the converter and the electronic commutator bridge, there is a low-pass filter made of inductance L1 and capacitors C1 and C2 C3 C4. The filter’s task is to supply the commutator bridge with low-pulsation DC voltage. In addition, the diagram shows the connection points of the LEM converter used to measure the current consumed by the electronic commutator bridge. Points marked in the diagram as GND1 GND2 GND3 with places of connection of three windings of the tested motor. The control of power transistors in a discrete manner (the transistor is fully conductive or completely blocked) is performed by a microcontroller. Due to the different potentials at the ends of the motor phases, the signal from the microcontroller must be galvanically isolated. In the implemented solution for this purpose, 6N136 optocouplers were used, which are shown in the diagram as U102, U202, U302, U402, U502, U602 for the transistors of the electronic commutator bridge (Figure 17), and the U702, U802 circuits for the DC/DC converter transistors (Figure 18). The output signal from the optocouplers is fed to the gates of power transistors through drivers dedicated to control MOSFET transistors. MAX627 circuits are used as drivers, characterized by the fact that one channel is inverting and the other is not. This feature was used by adding the possibility to feed the signal from one or the other driver’s channel to the transistor’s gate by switching the appropriate jumper. The power supply of individual drivers is provided by individual single watt voltage converters 24 V to 15 V type AM1S2415. These converters in the diagram are marked as U101, U201, U301, U401, U501, U601 for the electronic commutator bridge system (Figure 17), and U701, U801 for DC/DC converter transistors (Figure 18). The entire commutator system with the DC/DC converter was designed and made on one printed circuit board shown in the Figure 19 and its final realization is shown in the photo on the Figure 20. The designed board is of an evaluative nature. It was made with the possibility of conducting various types of research. There are additional terminals on the board and there is a possibility to connect additional additional elements.

**Figure 21:**
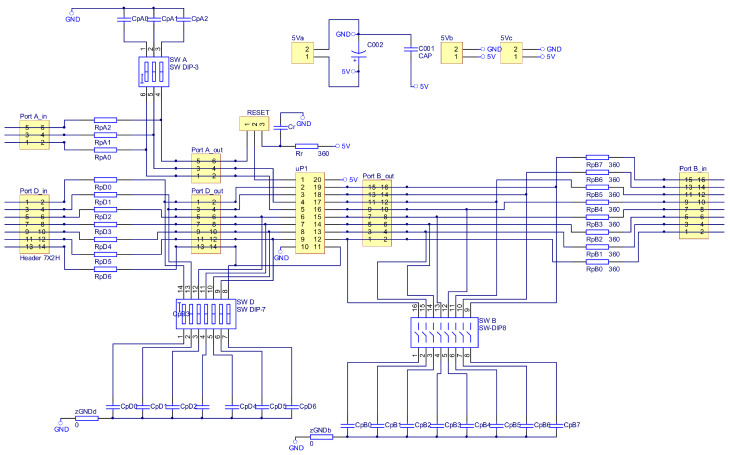
Schematic diagram of the construction of an electronic commutator with ATtiny2313 microcontroller.

**Figure 22:**
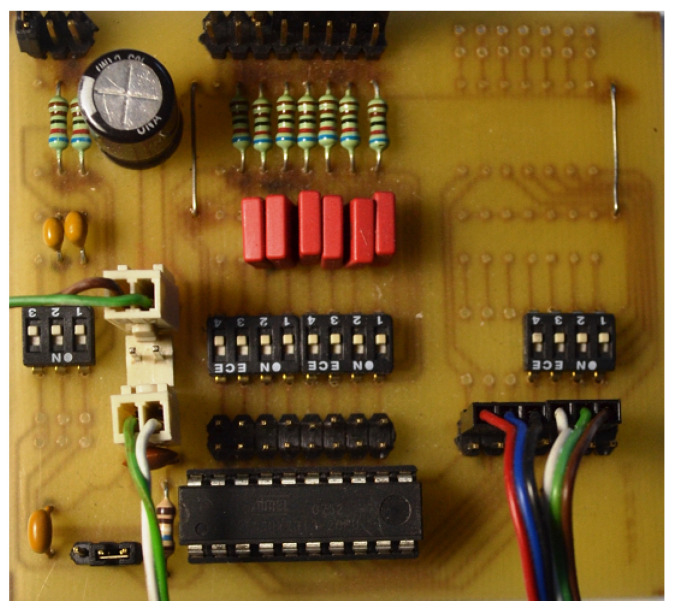
Electronic commutator circuit with ATtiny2313 microcontroller.

Additionally, as an independent element, a control board for transistors and DC/DC converter was designed and manufactured, the diagram of which is shown in Figure 21. This board was also made as an evaluation circuit allowing additional elements to be connected, for example to filter the signals supplied from the hall sensors. For an illustration of the PCB design, see [9]. The ready circuit dedicated to the tested solution is shown in the photo in Figure 22.

### 4.2. Power Transistors Control

The basic idea behind controlling power transistors is to switch the transistors sequentially based on the rotor position. The control process is discussed in detail in [9]. At this point, it should be noted that a very important aspect of control is the use of current limitations at the stage of engine start-up, reversal, braking and during engine operation. The idea of implementing the current limitation is quite simple and consists in the cyclical measurement of the current and then depending on the obtained result of regulating the voltage supplied to the motor windings. At this point, we will focus on discussing the motor reversing control.

**Figure 23:**
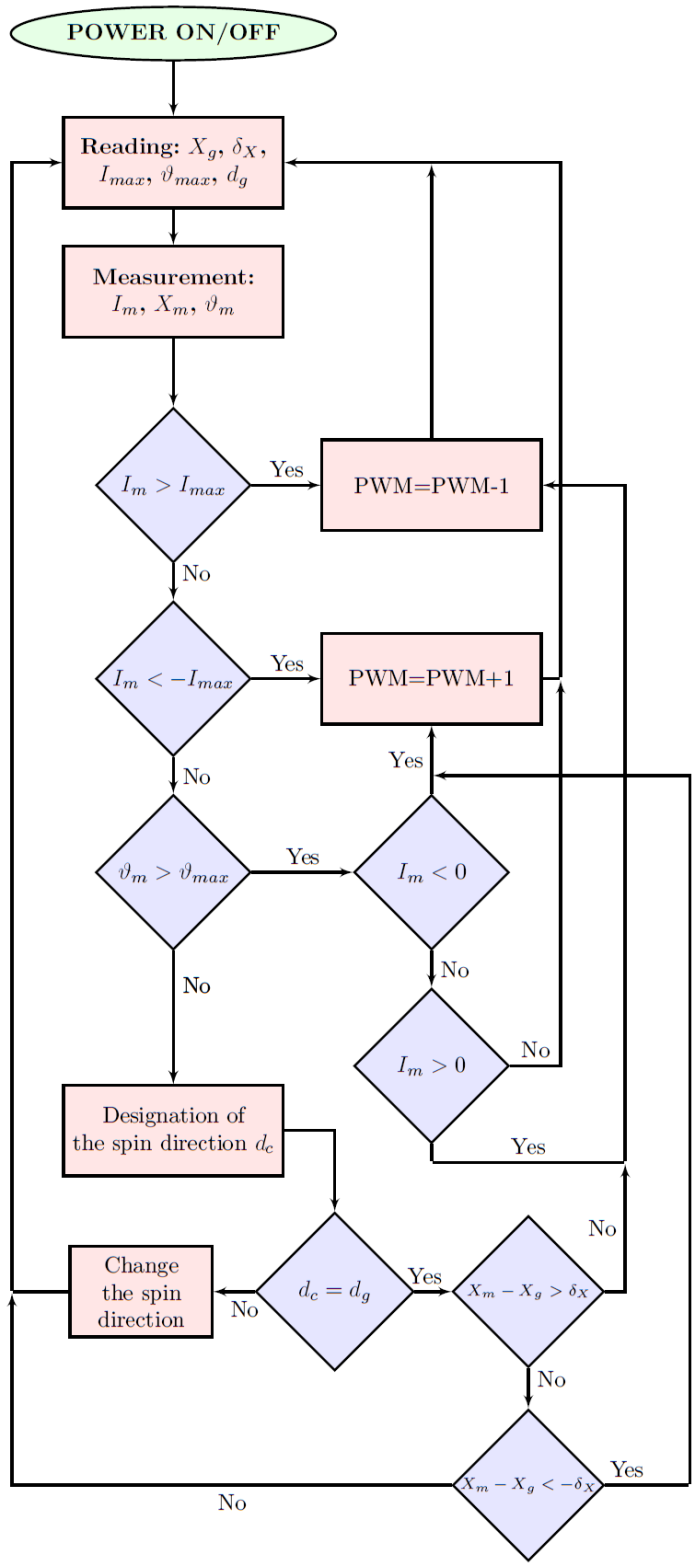
Block diagram of the microcontroller program which stabilizes the speed or the set mechanical torque *X* with current and temperature control.

**Figure 24:**
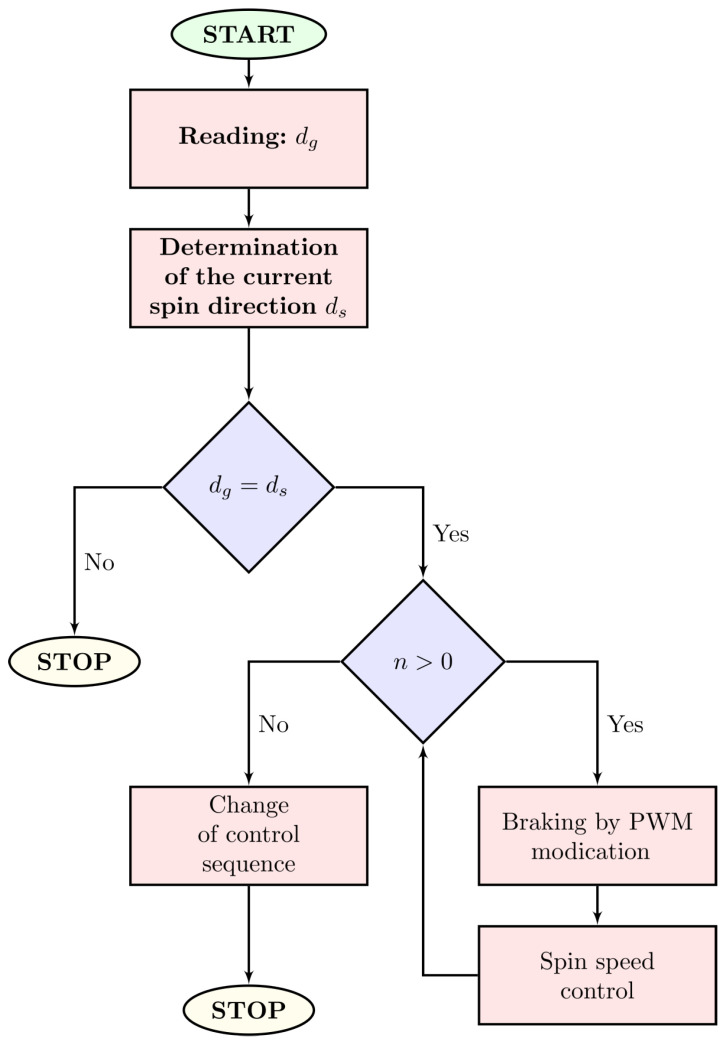
Block diagram of the microcontroller program responsible for the change of the direction of the motor rotation.

The reversal of the motor cannot be accomplished by simply driving the power transistors in the opposite direction of rotation. To realize the reversal of the BLDC motor, first reduce the speed until the rotor stops by switching the power transistors in the direction of rotation, and only then change the control sequence to the opposite. The speed reduction is preferably accomplished by regenerative braking by controlling the current. After changing the control sequence to the opposite, the motor is started with current control. The microprocessor controlling the electronic commutator implements the algorithm shown in Figure 23, where *X* indicates optionally: spin speed or driving torque depending on the choice of the control strategy. The $X_{g}$ parameter means the set value of the controlled quantity, $X_{m}$ the measured or its current value, $\delta_{X}$ the radius of the set value environment, $I_{max}$ the threshold current value natomias $I_{m}$—the measured current value, $\vartheta_{max}$—system temperature threshold value, $\vartheta_{m}$—measured temperature value, $d_{g}$—set spin direction, $d_{c}$—specific current spin direction. The algorithm block responsible for changing the spin direction is presented in detail in Figure 24.

**Figure 25:**
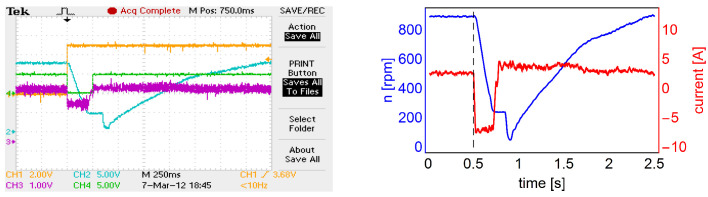
Oscillogram (**left** picture) shows the spin direction signal in orange, the spin direction signal in green, the recorded speed signal in blue, the motor current in purple, while the graph (**right** picture) shows the speed (blue line) and motor current (red line) and the dashed line indicates the start of a motor reversal.

An example of an electronic commutator operation during a reversal is shown in Figure 25. The included oscillogram shows the registration of four signals: pink—current, blue—motor rotation speed coming from the electronic tachogenerator, orange—set rotation direction and green compliance with the set rotation direction. The figure on the right shows the interpreted recording results, where the current is marked in red and the spin speed in blue. This figure shows that the current during braking has negative values, which proves the implementation of regenerative braking.

## 5. Research Results

The research was divided into two basic parts: the first one related to the study of the signals surrounding the motor shaft position and the second one related to the energy losses in the form of heat in the motor depending on the sensor displacement, which determines the position of the motor shaft and the method of powering the motor windings.

### 5.1. Comparison of External and Internal Sensor of the Motor Shaft Position

**Figure 26:**
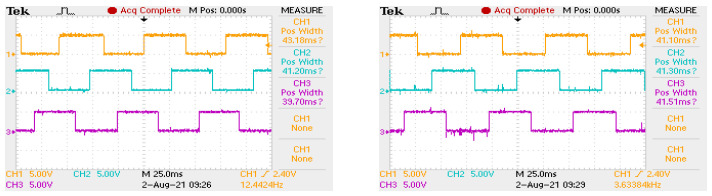
Oscillograms showing signals from Hall effect sensors installed in the engine (**left**) and from the outer target (**right**).

In this section, we want to discuss the comparison of external sensors (magnetic shield with Hall sensors) and internally arranged Hall sensors consists of examining the symmetry of the signals they provide. Sample oscillograms of signals coming from the hallotrons of the magnetic shield and hallhotrons placed in the grooves of the engine are shown in Figure 26. The oscillograms on the right side additionally show the duration of the high state of one signal period for each of the three hallotrons. In the case of the signal coming from hallotrons placed in the grooves of the engine, differences of the order of 10% can be seen.

Noting such a lack of symmetry in the duration of the halotron signal, it was decided to investigate this situation for the entire control sequence and refer to the results statistically. The control sequence consists of 6 different states represented by unique signal sequences from the three hallotrons. Due to the symmetry of the tested machine, the durations of individual states at a given predetermined rotational speed of the engine should be equal. Using the above assumption, a recorder was created that allows to record the duration of subsequent states. The recorder was made in such a way that the measurements were always performed for the same established sequence of states (recording always starts from a fixed position of the motor shaft, determined by an additional sensor). The recorder measures the duration of each of the states that make up one revolution of the motor shaft (three full control sequences). In addition, it is possible to record multiple times while the engine is running, making it possible to carry out multiple measurements for the same engine operating conditions.

The recorder is built on the basis of a microprocessor which, after one registration, sends data to the computer via a serial port. The registration is performed for a rotating motor with a fixed set speed and a constant load. The registration is then triggered by the operator. The recorded data are sent to the superior unit, where their statistical analysis is carried out (the given measurement attempt has been performed many times).

The symmetry of the signals determining the position of the engine rotor was tested for two motor work cases: first—the motor was driven by another motor (winding under test motor were not powered), the second—the motor was powered and kept the set speed. In first case studies were conducted for two different spin speeds: 340 [rpm] and 560 rot/min (due to the parameters of the drive motor). The results obtained in one series of measurements for the speed 340 [rpm] for individual states of the rotor position are presented in Table 2. Due to the fact that the same control states repeat every 1/3 revolution of the motor shaft, the times were compared for each of the parts constituting one full revolution.

In the second case, tests were conducted for three spin speeds: 340 [rpm], 680 [rpm], and rated speed 1000 [rpm]. The result of each measurement consists of 18 numbers representing the duration of each 18 states per revolution of the shaft engine. Each number represents the counted value of the microprocessor counter over the duration of one state. At a constant speed for a perfectly working system, the duration of all states will be the same, which will translate into registering 18 of the same numbers. In order to carry out analysis of measurement results, statistical calculations of duration times of individual states were made, making up one revolution of the motor shaft. Determined: arithmetic mean $\bar{x}$, the standard deviation of *s*, the volatility index *v*, and the relative maximum deviation from the mean *r*. Assuming that *k*-th number in the measurement result was denoted by $x_{k}$, the statistical indicators were defined as follows:

(1) $$\bar{x}=\frac{1}{n}\sum_{k=1}^{n}x_{k},$$

(2) $$\bar{s}=\sqrt{\frac{1}{n}\sum_{k=1}^{n}{\left(x_{k}-\bar{x}\right)}^{2}},$$

(3) $$\bar{v}=100\%\frac{s}{\bar{x}},$$

(4) $$\bar{r}=\max\left\{100\%\frac{\left|\bar{x}-x_{k}\right|}{\bar{x}},k=1,\cdots ,n\right\}.$$

The results of the statistical analysis for the case where the engine was powered by another machine are presented in Table 3. These data clearly show that the signals from hallotrons located in the nurseries differ significantly from the symmetry of their duration, in contrast to the signals from the outer target. Similar tests were carried out for the case where the engine spun at the set speeds and a fixed, unchanging load torque, as shown in Table 4.

The data presented in the tables clearly show that the use of an external magnetic disc provides signals determining the position of the motor shaft of much better quality than the sensors located in the motor slots. The lack of symmetry of signals from hallotrons in the slots of the motor may result from the fact that on the rotor circumference there are gaps between the glued magnets filled with steel forming the rotor shaft.

### 5.2. BLDC Motor Heating Test

As part of the research, a number of tests were carried out to heat the engine to a thermal steady state depending on:

- selection of sensors (external shield and hall sensors in slots) determining the switching moment of the power transistors of the electronic commutator bridge;
- choosing the ways of powering the motor windings.

The tests were carried out for selected setpoints of the motor load torque and selected setpoints of the spin speed. Due to the fact that the aim of the study is to compare which of the tested variants is characterized by the lowest losses of energy released in the form of heat in the engine, the engine fan was disassembled.

**Figure 27:**
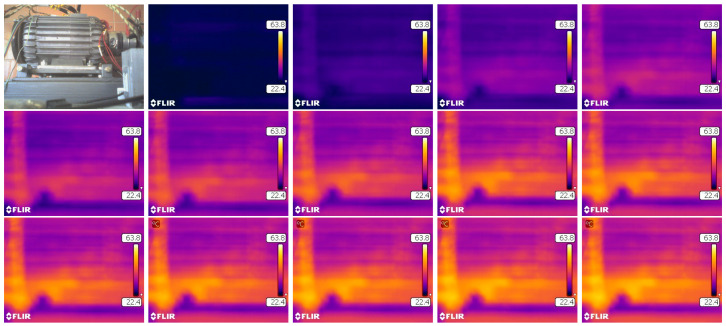
Research images from a thermal imaging camera heating applied to test our engine, photos are taken with time step Δt=15 min.

In each of the tested cases, the motor was heated to a steady thermal state, during which the temperature from Te1 and Te2 thermistors was recorded. An example of the engine heating process is shown in Figure 27. The photos shown here are taken from a thermal imaging camera. Subsequent registrations were performed with the same time step of 15 min.

The lack of a fan ensures comparable cooling conditions regardless of the engine spin speed. This approach allows us to determine qualitatively which of the tested variants is characterized by the lowest energy losses. The conducted tests showed that the lack of a fan made it impossible to test the BLDC motor at its rated parameters. In this case, excessive heating of the engine did not allow it to reach a thermally steady state.

**Figure 28:**
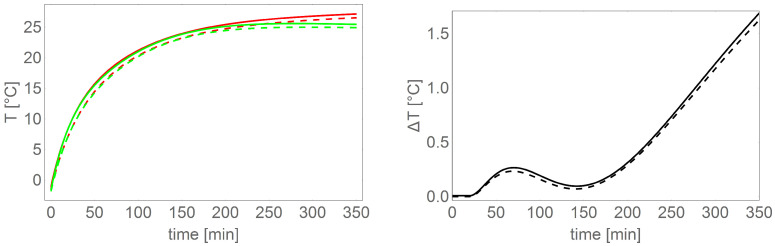
**Left** chart shows comparison of BLDC motor heating curves (Te1 dashed line, Te2 solid line) for two control variants: sensors in the motor (red), external magnetic disc (green), **right** chart shows differences between the heating temperatures shown on the **left** figure.

**Figure 29:**
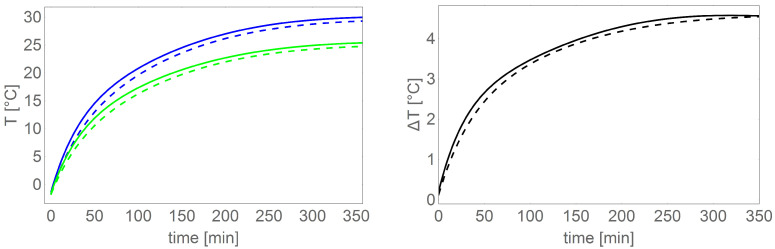
**Left** chart shows comparison of BLDC motor heating curves controlled based on an external sensor (Te1 dashed line, Te2 solid line) for two power supply variants: power supply without DC/DC converter (blue color), power supply via DC/DC converter (green), **right** chart shows differences between the heating temperature shown in the **left** picture.

Table 5 shows the results of motor heating (data from Te2) for the tested load torque and spin speed settings. Figure 28 and Figure 29 show a comparison of exemplary motor heating curves for operation with the same load torque and spin speed settings, with different control and power supply variants.

From the data in Table 5 and in the figures, it can be clearly concluded that the smallest energy losses in the motor separated in the form of heat are provided by the control variant based on an external magnetic disc and power supply based on a DC/DC converter.

The test results presented in Table 5 were obtained for one Pulse Width Modulation (PWM) equal to $f_{1}\approx 35\;\mathrm{kHz}$, $f_{2}\approx 18\;\mathrm{kHz}$, $f_{3}\approx 9\;\mathrm{kHz}$. It is known that the total losses in the motor change depend on the frequency of the PWM signal. The change of this parameter was not taken into account in the studies. The losses in the motor due to the frequency of the PWM signal will depend on the specific implementation, therefore our research focused on criteria independent of the BLDC motor design, in particular the materials used. The power supply and the BLDC motor control sequence designed as part of our research work does not require switching off the transistors in order to eliminate a short circuit. The bridge transistors are controlled in such a way that two transistors from two different branches are switched simultaneously. The delay in switching off the transistor will not short-circuit a given branch of the bridge, because the other transistors in this branch are turned off. Transistor switching times are incomparably shorter than the duration of a given control sequence. In addition, in the case of speed control with the use of a commutator bridge through PWM of the voltage controlling power transistors, there is also no possibility of a short circuit for a similar reason, because in a given branch the signal is modulated only for one transistor and at the same time due to the control sequence the other transistor is turned off.

## 6. Conclusions

As part of the research, a stand was built to ensure a given load torque for the BLDC motor and to ensure the given engine speed. For this purpose, it developed its own electronic commutator and prepared measuring equipment. Two different variants of the engine power supply and two different sensors for determining the position of the engine shaft have been proposed.

As part of the research, experiments were carried out to determine which of the sensors is better and which of the methods of powering the engine is more advantageous due to the energy losses in the engine released in the form of heat.

The conducted research has shown that the best solution is to use an external magnetic disc as a motor shaft position sensor and to use a DC/DC converter for speed regulation and current limiting functions. The engine shaft position sensor proposed in the paper was called external due to its novel implementation in our project. Of course, there is nothing to prevent such a sensor from being built inside the engine, but it would have to be taken into account at the engine design stage. However our proposed improvement is designed to be applied without changes to the construction of the BLDC motor. Moreover, such an external sensor concept could be built together with a suitably adapted fan and be an integral part of it if it is necessary for special use. The main advantage of this novel sensor mounting is the simplicity of construction and good precision in determining the positions of the motor rotor important for control. When it comes to the DC/DC converter, it is difficult to see application limitations due to the fact that the BLDC motor speed control requires a change in the average value of the voltage supplied to its windings. Our novel research has clearly shown that the use of a DC/DC converter produces less energy loss in the motor elements.

## Figures and Tables

**Table 1:** Selected BLDC motor applications and benefits of improved control for reduced energy losses in the form of heat in the motor.

Application	Limitations	Advances from Improved Control
road vehicle engines	weight, dimensions, energy consumption	lower energy consumption with the same traction parameters or more torque with the same vehicle weight or lower weight and dimensions with the same parameters of the driveline
ventilation and fans	motor size	smaller motor size with the same ventilation parameters or better ventilation with the same motor or the same size less energy loss or increased cooling efficiency (in the case of cooling systems)
servo motors	precise positioning, energy consumption	more precise positioning and lower energy consumption
power tools	energy consumption (battery powered), dimensions and weight	lower energy consumption with the same operating parameters or smaller size and weight ensuring the same operating parameters
drones, gliders and others	weight and energy consumption	range increase with the same parameters of the propulsion system

**Table 2:** Comparison of the duration times of individual commutation states determined by hall sensors installed in grooves and hall sensors installed in an external sensor for a rotating motor with a speed of 340 rpm.

State	Time [ms]					
	Hall Effect Sensors in Gaps			External Sensor		
	0–1/3 rot.	1/3–2/3 rot.	2/3–1 rot.	0–1/3 rot.	1/3–2/3 rot.	2/3–1 rot.
1	9.59	9.59	9.88	9.57	9.20	9.50
2	6.17	6.50	5.98	9.97	10.28	10.08
3	10.09	9.88	10.14	9.89	10.03	10.04
4	13.65	13.55	13.64	9.55	9.40	9.30
5	8.56	8.46	8.53	9.89	10.01	10.08
6	10.63	10.65	10.8	10.09	9.91	9.85
all	58.70	58.63	58.96	58.95	58.84	58.86

**Table 3:** Comparison of the symmetry of the signal determining the state of the rotor position for the hall effectors inside the engine and for the external sensor in the case of driving the engine.

rot. [1/min]	Hall Effect Sensors in Gaps				External Sensor			
	340		560		340		560	
Direction	0	1	0	1	0	1	0	1
$\bar{x}$ [ms]	9.79	9.73	6.19	6.16	9.82	9.83	6.17	6.17
*s* [ms]	3.754	2.399	2.328	1.49	0.413	0.378	0.245	0.260
*v* [%]	38.35	24.66	37.61	24.19	4.21	3.85	3.97	4.21
*r* [%]	72.17	68.93	71.38	67.76	10.80	9.12	9.99	9.69

**Table 4:** Comparison of the symmetry of the signal determining the state of the rotor position for the hall effect sensor inside the engine and for the external sensor in the case of idling.

rot. [1/min]	Hall Effect Sensors in Gaps						External Sensor					
	340		680		1000		340		680		1000	
Direction	0	1	0	1	0	1	0	1	0	1	0	1
$\bar{x}$ [ms]	9.79	8.85	4.88	4.48	3.30	2.75	9.56	9.78	4.82	4.87	3.34	3.32
*s* [ms]	4.03	3.75	1.91	2.13	1.3	1.59	0.35	0.34	0.19	0.15	0.12	0.09
*v* [%]	41.16	42.37	39.14	47.55	39.39	57.82	3.66	3.48	3.94	3.08	3.59	2.71
*r* [%]	70.08	91.27	67.25	90.67	74.63	107.31	7.65	6.99	6.96	6.42	7.27	5.88

**Table 5:** Summary of temperature increases to the steady state for selected moments of load and set centrifugation speeds, where $M_{N}$ is the rated torque of the motor and $n_{N}$ is the rated speed of the motor where the sensor *out* means the use of the external disk and the sensor in means the sensors placed in the engine for selected frequencies of the PWM signal: $f_{1}\approx 35\;\mathrm{kHz}$, $f_{2}\approx 18\;\mathrm{kHz}$, $f_{3}\approx 9\;\mathrm{kHz}$.

*M*	*n*	ΔT [${}^{\circ }$C]											
		Using DC/DC						PWM of Bridge					
		Sensor Out			Sensor In			Sensor Out			Sensor In		
		$f_{1}$	$f_{2}$	$f_{3}$	$f_{1}$	$f_{2}$	$f_{3}$	$f_{1}$	$f_{2}$	$f_{3}$	$f_{1}$	$f_{2}$	$f_{3}$
$1/3M_{N}$	$1/3n_{N}$	10.1	10.1	10.3	10.9	11	11.2	27.8	27.3	26.7	32.3	31.5	30.9
	$1/2n_{N}$	12.1	12.0	12.5	14.0	14.5	1 4.5	32.3	31.2	30.5	35.1	34.4	33.9
$1/2M_{N}$	$1/3n_{N}$	15.7	15.8	15.9	18.2	18.1	18.5	34.5	33.2	31.5	38.6	38,4	37.6
	$1/2n_{N}$	19.2	19.7	19.6	21.3	22.1	22.3	35.9	34.5	34.4	39.7	38.3	37.8
$2/3M_{N}$	$1/3n_{N}$	28.8	29.2	29.9	34.9	35.8	36.7	51.3	49.1	47.8	56.4	53.2	51.9
	$1/2n_{N}$	44.4	45.2	46.1	48.3	50.2	52.1	58.6	57.5	55.9	63.0	61.4	60.2
